# Quality of work‐life and coping strategies of nurse educators and clinicians in COVID‐19: A cross‐sectional study

**DOI:** 10.1002/nop2.1676

**Published:** 2023-02-22

**Authors:** Collins Atta Poku, Jonathan Bayuo, Eva Mensah, Victoria Bam

**Affiliations:** ^1^ Department of Nursing Kwame Nkrumah University of Science and Technology Kumasi Ghana; ^2^ School of Nursing and Midwifery University of Ghana Accra Ghana; ^3^ Department of Nursing Presbyterian University College Agogo Ghana; ^4^ Directorate of Nursing and Midwifery, Ghana Health Service Accra Ghana

**Keywords:** coping strategies, COVID‐19 pandemic, nurse clinicians and educators, quality of work‐life

## Abstract

The study compared perceived differences in Quality of Work‐Life (QoWL) among nurse clinicians and educators and coping strategies used by nurses.

**Design:**

A cross‐sectional study.

**Methods:**

From August and November 2020, the study measured the QoWL and coping strategies of 360 nurses with two scales using a multi‐stage sampling technique. The data were analysed with descriptive, Pearson correlation and multivariate linear regression analyses.

**Results:**

Quality of Work‐Life was generally low among nurses; nurse educators, however, had better QoWL than clinical nurses. Age, salary and nature of work predicted the QoWL of nurses. Work‐family segmentation, seeking assistance, open communication and recreational activities were employed by most nurses to cope with challenges. With the rate of workload and work‐related stress associated with COVID‐19, nurse leaders must advocate for evidence‐based coping strategies to deal with work and family life stress.

## 1 INTRODUCTION

In addressing the healthcare needs of the populace to ensure the attainment of the health‐related Sustainable Development Goals (SDGs), an improved Quality of Work‐Life (QoWL) of the health workforce plays a significant role. The nursing workforce in academia and clinical settings is central to the training of the nursing workforce of all categories and the provision of evidence‐based care to patients, respectively (Miles & Scott, 2019; Warshawsky & Cramer, 2019). While nurse educators have duple responsibilities of imparting nursing knowledge and providing other professional services to the populace, nurse clinicians provide preventive, curative, promotive, rehabilitative and palliative care to clients in the healthcare settings and the community. These tasks pose challenges to nurse educators with the increased student enrolment in various public nursing training institutions. Similarly, clinicians encounter stressful situations due to the challenges of increased workload and inadequacy of resources in the nursing practice environment (Al Kuwaiti & Subbarayalu, 2019; Koinis et al., 2015; Poku et al., 2020).

With the emergence of Coronavirus Disease (COVID‐19), there has been a record burden of morbidity and mortality among the nursing workforce especially nurses in clinical settings. This episode is exacerbated by inadequate personal protective equipment, fear of getting infected, and physical and emotional stress associated with the pandemic (Organization, W. H, 2020). The scope and load of work for the nursing workforce, both in the clinical and academia has also increased exponentially (Pourteimour et al., 2021), and this is a risk for diminished QoWL, which may affect the productivity of nurses (Shoja et al., 2020).

For academia, in the heat of COVID‐19, most low‐and‐middle‐income countries (LMICs) were confronted with a sudden disruption of planned academic activities, which was followed by virtual remote learning, with its associated challenges. Nurse educators suffered the brunt of the pandemic; key among the challenges was the inaccessibility of information technology infrastructure which negatively impacted virtual teaching, especially during the lockdown in most countries (Agu et al., 2021). Nurse educators working in areas of poor internet connectivity had to suffer the challenges of virtual teaching. Thus, there was a dilemma of teaching only students who had access to the virtual space or waiting for schools to reopen before teaching continued. The challenge of teaching practice‐based courses virtually for students to develop skills centred on clinical skills was also challenging for educators. The phenomenon is noted to have induced stress among nurse educators (Aslan & Pekince, 2021; Sheroun et al., 2020).

The nursing workforce in the clinical settings likewise experienced the challenge of ensuring a successful family–work interface, as they were confronted with ensuring that they do not infect their families with the coronavirus from the workplace (Agu et al., 2021; Costa et al., 2020). Consequently, the QoWL were reported to differ across the scope of work for nurse clinicians and educators. Though it has been reported that nurses who can cope well with changes brought by the COVID‐19 pandemic had higher scores on their QoWL (Keener et al., 2021; Rabacal et al., 2020), and anecdotally, there is a perception that nurse educators have higher QoWL compared with nurse clinicians. Nevertheless, nurses are encouraged to provide their respective roles to advance quality nursing care delivery (Inocian et al., 2021).

The concept of QoWL relates to the practice environment, the relationship with employers, working conditions, the perception of jobs, and organizational support systems. The QoWL stresses the need for improvement in the general welfare of the workforce (Scruth et al., 2018; Suleiman et al., 2019). Dealing with issues of QoWL is multifaceted, however, it is difficult to recognize the attributes that affect the QoWL in environments where much has not been done to decipher its influence on positive job outcomes (Akter et al., 2019; Fallahchai, 2022). It is worth noting that socio‐demographic status plays a major role in the QoWL of the workforce. In LMICs, the QoWL of nurses is low to moderate level, and the socio‐demographic status and work experience have been identified to significantly contribute to it (Albaqawi, 2018; Almalki et al., 2012). Additionally, it must be emphasized that the work environment can affect the way the workforce views their QoWL.

Policymakers have a unique role in solving the poor QoWL of the nursing workforce at all levels of organizations, as establishing work and life boundaries improves organizational and employee satisfaction (Akter et al., 2019; Albaqawi, 2018; Nayak & Sahoo, 2015). It is important to isolate the important elements that aid in developing a solution‐centred intervention to advance the QoWL nurses. Assessing nurses' perceptions of this concept is a major determinant for healthcare managers who are committed to improving patients and nursing job outcomes (Dehghan Nayeri, Salehi, Noghabi, & A., 2011; Els et al., 2021; Saygili et al., 2020).

In Sub‐Saharan Africa (SSA), Amin (2013) and Biresaw et al. (2020) report that nurses get fulfilment in some work factors that impact their QoWL. Nevertheless, it has been advocated that career advancement opportunities should be made available for nurses to ensure QoWL. Among other key factors to improve the QoWL of workers are a safe work environment, positive manager–subordinate relationships, and moderate workload; and this differs depending on the area of work (Dartey‐Baah, 2019; Roth et al., 2021). Meanwhile, stresses on nurses are reported to be aggravated by scarcity in the nursing workforce due to factors such as retirement, meagre wages, and limited assurance of better career prospects for nurses (Mason et al., 2013). Not only are nurses subjected to several job stressors, such as emotional exhaustion, stressful work schedules, and high workloads; they are unable to cope with these stressful situations, and this consequently affects their QoWL (Asiedu et al., 2018).

Ampofo and Dartey‐Baah (2016) ascertain that there is a significant positive influence of workers' QoWL on efficiency. It is cost‐effective for managers to ensure the QoWL of employees, as it improves quality work output. In effect, focusing on the overall satisfaction of staff by safeguarding their general welfare in the organization will lead to a positive outcome (Allan et al., 2019; Cummings et al., 2018; Wei et al., 2018).

In recent times, there has been a collective presumption that the attitude of nurses in hospitals towards patients and their significant others needs much to be desired; and one is tempted to attribute this unacceptable displacement of nurses' dissatisfaction with their QoWL (Asmaningrum et al., 2020; Kaddourah et al., 2018; Shakeri et al., 2021). While nursing leaders are making every effort to deliver standard care to the patient and improve patient satisfaction, it is important to lay down mechanisms to improve the welfare of the nursing workforce. This will not only enhance the public image of nursing but also can improve productivity and other organizational goals. It is also important to understand that poor QoWL of nurses can negatively impact nursing job outcomes, especially during the period of COVID‐19 and the nursing workforce shortage (Kelbiso et al., 2017).

A key challenge to low QoWL among nurses stems from the relative lack of formal work‐related regulations in most public institutions in SSA countries. This implies that managing work and other relative roles pose a bigger threat to nurses in SSA (Annor, 2016).

In overcoming the stressors or challenges associated with COVID‐19, work–family conflicts, increased workloads, unfriendly shift schedules, etc., establishing effective coping strategies or support systems is recommended. Though individuals in the past have engaged in numerous strategies to effectively cope with these work stressors, much cannot be said about SSA (Clark et al., 2014; McFadden et al., 2021). Though there may be different reasons for this development, there is a paucity of studies in Ghana which generally establish the differences between the QoWL among nurse clinicians and educators and their coping strategies in public health care settings. The study therefore aimed at comparing the perceived difference in QoWL among nurse clinicians and educators; the socio‐demographic and job characteristics that predict QoWL of nurses; and the coping strategies used by nurses in improving QoWL in public health facilities.

### 1.1 Research questions

1. What are the perceived differences in the QoWL among nurse educators and clinicians?
2. What factors predict the QoWL among nurses?
3. What coping strategies are used by nurses to improve QoWL?

## 2 METHODS AND MATERIALS

### 2.1 Study design and setting

A multicentre cross‐sectional approach, with a self‐administered questionnaire, was used to collect data on nurse clinicians and educators at public health facilities and health training institutions in one region in Ghana about QoWL. There are fourteen (14) government‐owned and quasi–Health Training institutions and twenty‐six (26) district hospitals in the region. Nine (9) health training institutions and five (5) hospitals were randomly selected for the study.

### 2.2 Participants

The population for the study was 676 nursing workforces (HRD of Ministry of Health, 2020): 435 clinicians and 241 educators. Only nurse clinicians with supervisory, administrative, and/or clinical roles and nurse educators with at least a rank of nursing officer were included in the study. Nurse clinicians and educators with less than 1 year of post‐qualification experience at their current job were excluded from the study.

### 2.3 Sample and sampling technique

Using Cochran's formula for sample estimation (*N* = z^2^ (pq)/e^2^) (Cochran, 1977), a total of 385 was estimated. The sample was adjusted by 10% due to potential attrition (Cohen et al., 2017); 424 participants – nurse clinicians (280) and nurse educators (144) were used for the study. A multi‐stage sampling approach was used for the study. All the centres (14) were randomly selected for the study. A proportionate stratified sampling method was used in assigning the sample to the 9 health training institutions and the 5 district hospitals based on each facility's numerical strength. Participants were conveniently sampled from each stratum.

### 2.4 Measures

#### 2.4.1 Socio‐demographic characteristics of participants

A questionnaire on socio‐demographic characteristics of nurse clinicians and educators including age, sex, marital status, educational status, rank, range of monthly income, work experience, working unit, and nature of work (clinical or education) was used.

#### 2.4.2 Quality of Work‐Life

*The Work‐Related Quality of Life Scale* (*WRQoL*) which is an evidence‐based measure of QoWL (Easton & Van Laar, 2018) was used. The WRQoL is a validated tool and measures a total of 23 items with six subscales (General Well‐Being—6 items, Home‐Work Interface—3 items, Job and Career Satisfaction—6 items, Control at Work—3 items, Working Conditions—3 items and Stress at Work—2 items). The tool is a 5‐point Likert scale with 1 (strongly disagree) to 5 (strongly agree). The total score of the scale is the sum of the scores of all items ‐ 23–73 (lower QoWL), 74–84 (average QoWL) and 85–115 (higher QoWL). The inter‐item consistency of the scale was measured by Cronbach's alpha with a value of 0.91 (Easton & Van Laar, 2018). The questionnaire was pre‐tested with 20 participants at a Government Hospital to ascertain the content validity of the measures, and Cronbach's alpha was 0.81. Other studies have also reported an acceptable reliability coefficient (Duracinsky et al., 2022; Howie‐Esquivel et al., 2022; McFadden et al., 2021; Pereira et al., 2021).

#### 2.4.3 Coping strategy

Clark et al's *Work Stressor Coping Scale* was used to measure the coping of nurses (Clark et al., 2014). The 36‐items scale assesses 12 coping strategies. The scale is on a 6‐point Likert scale of ‘Never have done this’—1 and ‘Almost always do this’—6. Each coping strategy is represented by three items, and a mean score is calculated for each coping strategy. The score of the scale is determined by the sum of the frequencies of items, with higher scores indicating the increased frequency of using a specific coping strategy. The scale has a good internal consistency with Cronbach's alpha of 0.83. The scale also reports good evidence on both convergent and discriminant validity; and has been used in other studies (Clark et al., 2014).

### 2.5 Data collection

This study sought ethical approval (REDACTED). Data collection was undertaken between August and November 2020. The participants were contacted in person to enlist their participation and to plan the date for the administration of questionnaires. The paper‐based questionnaires were sent to the nursing managers of the various facilities, from where it was distributed to the participants, following written consent. Confidentiality and anonymity were assured. Participants were also informed about their right to withdraw from the study at any point in time without any repercussions. Questionnaires were filled out independently and returned the same day.

### 2.6 Statistical analysis

Data were analysed using IBM SPSS Statistics (Version 26.0). Descriptive statistics were used to present the socio‐demographic and job characteristics, QoWL and coping strategies of the participants. An independent t‐test was used to compare the differences in the mean score of nurse clinicians and educators on their QoWL. Pearson's moment product correlation analysis was used to explore the relationship between socio‐demographic characteristics and QoWL. Multivariate regression analysis was conducted on the predictors (age, salary level, years of service, and nature of work) on the QoWL of nurses as a dependent variable. Initial analysis was done to safeguard no violation of the assumptions of linearity, data normality, homoscedasticity, and multicollinearity. The analysis was conducted at a 95% confidence level.

## 3 RESULTS

### 3.1 Socio‐demographic characteristics, QoWL and coping strategies score of participants

The study estimated a response rate of 84.9% with a total sample of 360 participants. The results on the socio‐demographic characteristics of participants are summarized in Table 1. The participants had a mean age of 39 ± 4.89 years. With regards to gender, it was dominated by females (78.9%, *n* = 284). Participants with a bachelor's degree as their highest qualification were the majority (52.2%, *n* = 188) with most of them being clinicians (64.4%, *n* = 232). The results also revealed a low mean score for overall QoWL among nurses in the study (Mean = 56.92 ± 13.33); with a greater proportion (53.9%, *n* = 194) rating their overall QoWL as low. While nurses coped well with the QoWL (mean = 67.35, SD = 5.12), most of them effectively used the work–family segmentation coping strategy (mean = 14.91 ± 3.19).

**TABLE 1:** Socio‐demographic characteristics of participants in the study.

Variable	Frequency (*N* = 360)	Per cent (%)	Mean (SD)
Age			39 (4.89)
Gender			
Male	76	21.1	
Female	284	78.9	
Marital status			
Single	105	29.2	
Married	247	68.6	
Separated	5	1.4	
Widowed	3	0.8	
Living with spouse			
Yes	184	74.5	
No	63	25.5	
Highest qualification			
Diploma	64	17.8	
Bachelor	188	52.2	
Masters	108	30.0	
Years of practice			
Below 5 years	156	43.3	
5–10 years	162	45.0	
Above 10 years	42	11.7	
Monthly salary (US$)			
Below 350	54	15.0	
350–700	231	64.2	
Above 700	75	20.8	
Nature of work			
Clinical	232	64.4	
Education	128	35.6	
Perceived QoWL of nurses			56.92 (13.33)
Clinicians			
Lower	100	27.8	
Average	88	24.4	
Higher	44	12.2	
Educators			
Lower	55	15.3	
Average	49	13.6	
Higher	24	6.7	
Coping strategies			67.35 (5.12)
Seeking assistance/sharing the workload			12.60 (1.83)
Being organized/planning/ scheduling			12.44 (1.75)
Verbalizing with others/Communication			13.84 (1.86)
Work–family segmentation			14.91 (3.19)
Recreation and relaxation			13.56 (2.10)

### 3.2 Comparison of quality of work‐life between nurse clinicians and educators

An independent sample *t*‐test was conducted to compare the QoWL of clinicians and educators as shown in Table 2. The findings revealed a statistically significant difference in the overall QoWL between clinicians and educators [*t* (358) = −5.432, *p* < 0.001]; with educators having higher QoWL (*M* = 73.20, SD = 13.17) than clinicians (*M* = 66.56, SD = 12.86). Regarding the various domains of QoWL, the results revealed a significant difference in the QoWL on general wellbeing [*t* (358) = −5.432, *p* < 0.001]; with educators having the higher QoWL (*M* = 19.69, SD = 4.00) compared with their counterparts in the clinical setting (*M* = 16.93, SD = 4.92). Additionally, there was a statistically significant difference in the mean scores on working conditions [*t* (358) = −6.987, *p* > 0.001]; where educators had the highest mean score (*M* = 8.84, SD = 2.81) than clinicians (*M* = 6.70, SD = 2.76). Though there was no significant difference in QoWL with regards to homework interface [*t* (358) = −1.45, *p* = 0.148], and job career satisfaction [*t* (358) = −1.44, *p* = 0.150], educators had higher QoWL than clinicians on those domains. Conversely, clinicians had a higher mean score on stress at work (*M* = 6.51, SD = 1.99) when compared with educators (*M* = 6.31, SD = 2.21). Despite the non‐existence of significant differences [*t* (358) = 0.880, *p* = 0.380], the results imply that clinicians experience higher levels of stress than educators.

**TABLE 2:** Comparison of Quality of Work‐Life between nurse clinicians and educators.

QoWL domain	Nature of work	Level of QoWL			Mean	SD	T	df	Sig.
		Lower	Average	Higher					
General Wellbeing	Clinical	100	88	44	16.94	4.92	−5.432	358	0.000
	Education	55	49	24	19.70	4.00	−5.763	309.00	0.000
Home Work Interface	Clinical	138	55	39	8.47	2.86	−1.451	358	0.148
	Education	75	31	22	8.90	2.40	−1.526	302.07	0.128
Job and Career Satisfaction	Clinical	79	68	85	20.07	4.75	−1.443	358	0.150
	Education	44	38	46	20.84	4.97	−1.424	251.83	0.156
Control at Work	Clinical	133	48	51	7.88	3.16	−2.112	358	0.035
	Education	73	27	28	8.63	3.23	−2.099	257.03	0.037
Working Conditions	Clinical	178	48	6	6.70	2.76	−6.987	358	0.000
	Education								
		98	16	4	8.84	2.80	−6.957	258.67	0.000
Stress at Work	Clinical	76	79	77	6.51	1.99	0.880	358	0.380
	Education	41	44	43	6.31	2.21	0.853	239.16	0.395
Overall QoWL	Clinical	100	88	44	66.56	12.86	−4.648	358	0.000
	Education	55	49	24	73.20	13.17	−4.617	256.79	0.000

### 3.3 The correlation between the socio‐demographic characteristics and QoWL of nurses

The relationship between the QoWL and its domains as measured by the WRQoL scale and the socio‐demographic characteristics of the nurse was investigated using Pearson product–moment correlation coefficient. There was a weak positive correlation between the QoWL of the nurse and all the socio‐demographic variables except the qualification of the nurse as follows; age (*r* = 0.120, *p* < 0.05), years of services as a nurse (*r* = 0.190, *p* < 0.001), and salary level (*r* = 0.239, *p* < 0.001). The age and salary of the nurse correlated positively with the domains of QoWL stress at work as detailed in Table 3.

**TABLE 3:** Correlations between socio‐demographic characteristics and QoWL.

Variables	1	2	3	4	5	6	7	8	9	10	11
Age	‐										
2 Years of service	0.884**	‐									
3 Qualification	0.508**	0.535**	‐								
4 Salary	0.579**	0.612**	0.523**	‐							
5 GWB	0.152**	0.227**	0.143**	0.172**	‐						
6 HWI	0.152**	0.210**	0.010	0.216**	0.337**	‐					
7 JCS	0.037	0.091	−0.074	0.111*	0.366**	0.513**	‐				
8 CAW	0.119*	0.145**	−0.039	0.185**	0.230**	0.489**	0.660**	‐			
9 WC	0.115*	0.162**	0.200**	0.248**	0.304**	0.533**	0.480**	0.385**	‐		
10 SAW	−0.157**	−0.161**	−0.040	0.008	0.009	−0.228**	−0.017	−0.004	−0.066	‐	
11 QoWL	0.120*	0.190**	0.046	0.239**	0.630**	0.701**	0.852**	0.744**	0.685** ^y^	0.095	‐

*Note*: Independent variable: CAW, Control at Work; GWB, General Well‐Being; HWI, Home‐Work Interface; JCS, Job and Career and Satisfaction; QoWL, Quality of Work‐Life; SAW, Stress at Work; WC, Working Conditions.

* = Correlation is significant at 0.05 level (2‐tailed).

** = Correlation is significant at 0.01 level (2‐tailed).

### 3.4 The predictive effects of socio‐demographic characteristics on QoWL

A linear regression analysis was conducted to determine the predicting effects of the demographic characteristics of the nurse (age, salary level and nature of work) on the QoWL of nurses as presented in Table 4. After the entry of the socio‐demographic data, the total variance explained by the model as a whole was 8.0% [F _(3, 355)_ = 10.26, *p* < 0.05]. In the model, all demographic characteristics had a positive statistically significant relationship with the QoWL of the nurse with the salary level of the nurse (β = 0.233, *p* < 0.05) recording the highest value (β = 0.269, *p* < 0.05), followed by nature of work (β = 0.152, *p* < 0.05). The age of the nurse, however, had a negative statistically significant relationship with the QoWL of the nurse (β = −0.041, *p* < 0.05).

**TABLE 4:** Predictors of Quality of Work‐Life among nurses.

Variables		B	Std. error	Beta	T	Sig.
	(Constant)	79.813	7.365		10.836	0.000
	Age	−0.106	0.163	−0.041	−0.654	0.040
	Salary level	4.634	1.251	0.233	3.704	0.000
	Nature of work	0.959	0.328	0.152	2.922	0.004
Model summary: *R* ^*2*^ = 0.08, F _(3, 355) =_10.26, *p* < 0.05						

Dependent variable: Overall quality of work‐life (QoWL).

## 4 DISCUSSION

The study sought to compare the perceived differences between QoWL among nurse clinicians and educators in facilities in the middle belt of Ghana. The study findings suggest that nurse educators have higher QoWL than clinical nurses. Also, regarding stress, nurse clinicians scored comparatively higher than nurse educators. Age, salary level and nature of work were the main predictors of QoWL among both categories of nurses. Overall, the study findings highlight that the clinical setting is a major source of stress when compared with the classroom warranting a need to consider strategies for attenuating these stressors.

Quality of Work‐Life is a complex, multidimensional concept involving work, persons and organizations. A person's QoWL is influenced by several factors, including work tension, work environment and organizational policies. Now more than ever, the issue of QoWL has emerged as a critical issue considering its effect on nurses' turnover intention. In the current study, it was observed that the majority of the nurses had low to average QoWL, with most being nurse clinicians. Additionally, nurse clinicians were observed to experience more stress than nurse educators. Potentially, these interesting findings may be related to the different work environments and organizational climates. The clinical environment, depending on the unit, can be chaotic with patients having varying acuity levels which may increase the level of stress experienced by nurse clinicians in leadership or managerial roles (Poku et al., 2022). While there is currently no study in the Ghanaian context exploring this, studies involving clinical nurses in units such as the burn intensive care unit (Bayuo, 2018; Bayuo & Agbenorku, 2018) and emergency department (Poku et al., 2020; Xu et al., 2019) have underscored the presence of high levels of stress emanating from the increasing patient caseloads, treatment processes, unpredictable nature of care, often with limited to inadequate professional support for staff. Even though some nurse clinicians do not offer direct clinical care, they may be predisposed to stress as they offer leadership in their respective units. Nurse leaders need to deal with staff shortages, poor working conditions, inadequate management support, and heavy workload (Ofei et al., 2020). With the emergence of COVID‐19, clinical nurses on the frontline need to deal with increasing levels of stress and other psychosocial issues (Labrague & de Los Santos, 2021; Murat et al., 2021; Shahrour & Dardas, 2020). Several studies have reported increasing levels of burnout and fatigue among frontline nurses emanating from the nature of the novel infection, high mortality rates, and fear of contracting the infection and spreading it to one's relatives (Arnetz et al., 2020; Lorente et al., 2021; Mo et al., 2020). Contracting COVID‐19 will also require self‐quarantine implying that nurse managers need to deal with more issues regarding staff shortages and heavy workload (Dimino et al., 2021; Labrague & de Los Santos, 2021). These escalating concerns may have contributed to the stress experienced by the nurse managers in the current study.

The aforementioned assertions are not a claim that the educational setting is exempt from work‐related stress. Nurse educators usually have the additional responsibility of the academic advisor to students. Students, for that matter, often engage educators in private conversations. The mentor or advisor role by educators opens an avenue of latent disclosure anxiety, stress and trauma experienced by students which can contribute to the development of burnout and compassion fatigue (Branch et al., 2011). Taken together, these findings should direct attention to developing context‐specific support structures commensurate to nurses' needs. Support measures such as offering avenues for release, mindfulness, and peer support may be considered. These are essential as it has been established that nurses' QoWL results in positive patient outcomes from safe and quality healthcare (McMurray, 2020).

Previous studies have established that factors such as work environment, work conditions, work stress, and psychosocial factors may predict a clinician's QoWL (Horrigan, 2017; Horrigan et al., 2013). In the current study, age, years of service, salary level and primary specialty emerged as predictors of QoWL; with years of service being the most significant predictor. The age of the healthcare practitioner is generally recognized as a significant predictor of burnout and compassion fatigue which can affect one's QoWL (Gribben & Semple, 2021; Lee et al., 2015). Considering a mean age of 39 years observed in this study, it reflects relatively young nurse managers. This finding suggests that younger nurses may be at a higher risk for poor QoWL which strengthens the need for ongoing peer support from other senior colleagues. Regarding years of service and remuneration, it has been established that an increasing number of working years and higher remuneration may be associated with lower burnout which may translate to increased QoWL and vice versa (Kamal et al., 2020). Thus, new staff members are a higher risk group, particularly in the absence of workplace support. This finding may be related to the fact that an increasing number of years may come along with identifying effective coping strategies and mechanisms to thrive in a clinical area, which again may affirm the indispensable roles played by senior staff's QoWL (McFadden et al., 2021). By using the Job Demands‐Resource model, researchers in the past have advocated for the provision of resources as a buffer in promoting QoWL (Bakker & Demerouti, 2007; Schaufeli & Taris, 2014). The findings of this study succinctly agreed with the use of recreational activities, seeking assistance from colleagues and managers, disengagement of work and family roles and prior planning or scheduling of work to reduce stress.

### 4.1 Limitations

The strength of this study lies in the multicentre approach involving nurse managers and nurse educators. This approach provides a fuller understanding of the phenomenon. Despite this, some limitations are noteworthy. First, the data only provides a quantitative evaluation of the phenomenon but does not offer any insight as to why and how these may occur. Thus, future studies may need to consider a mixed or multi‐method approach. Second, the data were collected at a time when the country was battling the COVID‐19 pandemic which may have had some repercussions on clinical nurses. A follow‐up study post‐pandemic is therefore recommended. Future research could also focus on the work–family conflict with the changing dynamics in the healthcare system; thus, undertaking a study that will provide an understanding of organizational initiatives that can improve the job outcomes of the nursing workforce. Moreover, the reality of the nursing profession as women‐dominated was reflected in findings as close to 80% of the study participants were females. This is similar to nursing workforce data in Ghana. The possibility of bias in reporting the female voice was high. Conscious efforts were therefore made by the researchers to at least have the 20% of the respondents be males to be able to have a balance concerning the sex distribution of the nursing workforce.

## 5 CONCLUSION

With the increasing rate of workload and work‐related stress associated with COVID‐19, healthcare managers must reassess the QoWL of nurses and advocate evidence‐based coping strategies that will help in dealing with the stress associated with both work and family life. These may include seeking assistance from managers and co‐workers, encouraging nurses to disengage work roles from family responsibilities, effective team communication and support, and recreational activities to reduce stress thereby improving the QoWL of nurses. Other recommended initiatives for policy or healthcare managers may include investing in creating a professional practice environment with an improved work climate, good remuneration and flexible work schedule to improve the QoWL of the nursing workforce, and its effect will enhance other nursing job outcomes such as job satisfaction and decreased turnover rate.

## AUTHOR CONTRIBUTIONS

CAP, EM and JB conceptualized and designed the study method. CAP, VAA and JB carried out the data collection, analysis and interpretation of data. CAP, EM and VB originally drafted the manuscript. All authors read, revised and approved the final manuscript for submission.

## FUNDING INFORMATION

No funding was received for this study.

## CONFLICT OF INTEREST STATEMENT

The authors declare that they have no competing interests.

## RESEARCH ETHICS COMMITTEE APPROVAL

Ethical approval was sought from the Komfo Anokye Teaching Hospital Institutional Review Board with the number KATH IRB/AP/094/20 while participants also gave verbal consent before data collection as approved by the IRB.

## CONSENT FOR PUBLICATION

All authors have approved the manuscript for submission.

## Data Availability

The data that supports the findings of this study are available in the supplementary material of this article.

## References

[nop21676-bib-0001] Agu, C. F. , Stewart, J. , McFarlane‐Stewart, N. , & Rae, T. (2021). COVID‐19 pandemic effects on nursing education: Looking through the lens of a developing country. International Nursing Review, 68(2), 153–158. 10.1111/inr.12663 33513283 PMC8014519

[nop21676-bib-0002] Akter, N. , Akter, M. K. , & Turale, S. (2019). Barriers to quality of work life among Bangladeshi nurses: A qualitative study. International Nursing Review, 66(3), 396–403. 10.1111/inr.12540 31393005

[nop21676-bib-0003] Al Kuwaiti, A. , & Subbarayalu, A. V. (2019). Health sciences teaching staff's perception about quality of work life in saudi universities: Reliability and validity of the questionnaire instrument. Hamdan Medical Journal, 12(4), 182. 10.4103/HMJ.HMJ_87_18

[nop21676-bib-0004] Albaqawi, H. M. (2018). Quality nursing work life among nurses in hail region, Kingdom of Saudi Arabia: Redefining the boundaries of work and life. Advances in Social Sciences Research Journal, 5(3), 433–439. https://doi.org/1014738/assrjj.53.4341

[nop21676-bib-0005] Allan, B. A. , Batz‐Barbarich, C. , Sterling, H. M. , & Tay, L. (2019). Outcomes of meaningful work: A meta‐analysis. Journal of Management Studies, 56(3), 500–528. 10.1111/joms.12406

[nop21676-bib-0006] Almalki, M. J. , FitzGerald, G. , & Clark, M. (2012). Quality of work life among primary health care nurses in the Jazan region, Saudi Arabia: A cross‐sectional study. Human Resources for Health, 10(1), 30. 10.1186/1478-4491-10-30 22971150 PMC3543175

[nop21676-bib-0007] Amin, A. M. (2013). Assessment of Quality of Work Life of Nurses in Tamale Teaching Hospital [PhD Thesis]. University of Ghana.

[nop21676-bib-0008] Ampofo, E. Y. , & Dartey‐Baah, K. (2016). Missing link between quality of work life and productivity of loan disbursement: The Ghanaian perspective. International Journal of Business and Management, 11(3), 203. 10.5539/ijbm.v11n3p203

[nop21676-bib-0009] Annor, F. (2016). Work–family demands and support: Examining direct and moderating influences on work–family conflict. Journal of Workplace Behavioral Health, 31(2), 87–103. 10.1080/15555240.2015.1119656

[nop21676-bib-0010] Arnetz, J. E. , Goetz, C. M. , Arnetz, B. B. , & Arble, E. (2020). Nurse reports of stressful situations during the COVID‐19 pandemic: Qualitative analysis of survey responses. International Journal of Environmental Research and Public Health, 17(21), 8126. 10.3390/ijerph17218126 33153198 PMC7663126

[nop21676-bib-0011] Asiedu, E. E. A. , Annor, F. , Amponsah‐Tawiah, K. , & Dartey‐Baah, K. (2018). Juggling family and professional caring: Role demands, work–family conflict and burnout among registered nurses in Ghana. Nursing Open, 5(4), 611–620. 10.1002/nop2.178 30338107 PMC6178356

[nop21676-bib-0012] Aslan, H. , & Pekince, H. (2021). Nursing students' views on the COVID‐19 pandemic and their percieved stress levels. Perspectives in Psychiatric Care, 57(2), 695–701. 10.1111/ppc.12597 32808314 PMC7461415

[nop21676-bib-0013] Asmaningrum, N. , Kurniawati, D. , & Tsai, Y.‐F. (2020). Threats to patient dignity in clinical care settings: A qualitative comparison of Indonesian nurses and patients. Journal of Clinical Nursing, 29(5–6), 899–908. 10.1111/jocn.15144 31855306

[nop21676-bib-0014] Bakker, A. B. , & Demerouti, E. (2007). The job demands‐resources model: State of the art. Journal of Managerial Psychology, 22, 309–328. 10.1108/02683940710733115

[nop21676-bib-0015] Bayuo, J. (2018). Nurses' experiences of caring for severely burned patients. Collegian, 25(1), 27–32. 10.1016/j.colegn.2017.03.002

[nop21676-bib-0016] Bayuo, J. , & Agbenorku, P. (2018). Coping strategies among nurses in the burn intensive care unit: A qualitative study. Burns Open, 2(1), 47–52. 10.1016/j.burnso.2017.10.004

[nop21676-bib-0017] Biresaw, H. , Boru, B. , & Yimer, B. (2020). Quality of nursing work life and associated factors in Amhara region referral hospitals, Northwest Ethiopia: A cross sectional study. International Journal of Africa Nursing Sciences, 13, 100214. 10.1016/j.ijans.2020.100214

[nop21676-bib-0018] Branch, K. A. , Hayes‐Smith, R. , & Richards, T. N. (2011). Professors' experiences with student disclosures of sexual assault and intimate partner violence: How “helping” students can inform teaching practices. Feminist Criminology, 6(1), 54–75. 10.1177/1557085110397040

[nop21676-bib-0019] Clark, M. A. , Michel, J. S. , Early, R. J. , & Baltes, B. B. (2014). Strategies for coping with work stressors and family stressors: Scale development and validation. Journal of Business and Psychology, 29(4), 617–638. 10.1007/s10869-014-9356-7

[nop21676-bib-0020] Cochran, W. G. (1977). Sampling Techniques. John Wiley & Sons.

[nop21676-bib-0021] Cohen, L. , Manion, L. , & Morrison, K. (2017). Surveys, longitudinal, cross‐sectional and trend studies. In Research methods in education (pp. 334–360). Routledge.

[nop21676-bib-0022] Costa, R. , Lino, M. M. , Souza, A. I. J. d. , Lorenzini, E. , Fernandes, G. C. M. , Brehmer, L. C. d. F. , Vargas, M. A. d. O. , Locks, M. O. H. , & Gonçalves, N. (2020). Nursing teaching in covid‐19 times: How to reinvent it in this context? In Texto & Contexto‐Enfermagem (Vol. 29). SciELO Brasil. 10.1590/1980-265X-TCE-2020-0002-0002

[nop21676-bib-0023] Cummings, G. G. , Tate, K. , Lee, S. , Wong, C. A. , Paananen, T. , Micaroni, S. P. , & Chatterjee, G. E. (2018). Leadership styles and outcome patterns for the nursing workforce and work environment: A systematic review. International Journal of Nursing Studies, 85, 19–60. 10.1016/j.ijnurstu.2009.08.006 29807190

[nop21676-bib-0024] Dartey‐Baah, K. (2019). Leadership styles and workplace wellness among Ghanaian SME workers. International Conference on Applied Human Factors and Ergonomics, 961, 561–572.

[nop21676-bib-0025] Nayeri, N. D. , Salehi, T. , & Noghabi, A. A. (2011). Quality of work life and productivity among Iranian nurses. Contemporary Nurse, 39(1), 106–118. 21955271 10.5172/conu.2011.39.1.106

[nop21676-bib-0026] Dimino, K. , Learmonth, A. E. , & Fajardo, C. C. (2021). Nurse managers leading the way: Reenvisioning stress to maintain healthy work environments. Critical Care Nurse, 41(5), 52–58. 10.4037/ccn2021463 33647958

[nop21676-bib-0027] Duracinsky, M. , Marcellin, F. , Cousin, L. , Di Beo, V. , Mahé, V. , Rousset‐Torrente, O. , Carrieri, P. , & Chassany, O. (2022). Social and professional recognition are key determinants of quality of life at work among night‐shift healthcare workers in Paris public hospitals (AP‐HP ALADDIN COVID‐19 survey). PLoS One, 17(4), e0265724. 10.1371/journal.pone.0265724 35390061 PMC9045406

[nop21676-bib-0028] Easton, S. , & Van Laar, D. (2018). User manual for the work‐related quality of life (WRQoL) scale: A measure of quality of working life (2nd ed.). University of Portsmouth. 10.17029/EASTON2018

[nop21676-bib-0029] Els, V. , Brouwers, M. , & Lodewyk, R. B. (2021). Quality of work life: Effects on turnover intention and organisational commitment amongst selected south African manufacturing organisations. SA Journal of Human Resource Management, 19, 1407.

[nop21676-bib-0030] Fallahchai, R. (2022). Occupational stress, dyadic adjustment and quality of work‐life in married nurses: Moderating effects of dyadic coping. International Journal of Nursing Practice, 28(1), e13032. 10.1111/ijn.13032 34935250

[nop21676-bib-0031] Gribben, L. , & Semple, C. J. (2021). Prevalence and predictors of burnout and work‐life balance within the haematology cancer nursing workforce. European Journal of Oncology Nursing, 52, 101973. 10.1016/j.ejon.2021.101973 34015591

[nop21676-bib-0032] Horrigan, J. M. (2017). Evaluating the Quality of Work Life of Registered Nurses in Urban, Rural and Remote Northeastern Ontario [PhD Thesis]. Laurentian University of Sudbury.

[nop21676-bib-0033] Horrigan, J. M. , Lightfoot, N. E. , Larivière, M. A. , & Jacklin, K. (2013). Evaluating and improving nurses' health and quality of work life: A cross‐sectional study of Korean blue collar workers employed by small businesses. Workplace Health & Safety, 61(4), 173–181. 10.1177/216507991306100405 23557346

[nop21676-bib-0034] Howie‐Esquivel, J. , Do Byon, H. , Lewis, C. , Travis, A. , & Cavanagh, C. (2022). Quality of work‐life among advanced practice nurses who manage care for patients with heart failure: The effect of resilience during the Covid‐19 pandemic. Heart & Lung, 55, 34–41. 10.1016/j.hrtlng.2022.04.005 35447467 PMC8995301

[nop21676-bib-0035] HRD of Ministry of Health . (2020). Annual Report for 2019 .

[nop21676-bib-0036] Inocian, E. P. , Cruz, J. P. , Saeed Alshehry, A. , Alshamlani, Y. , Ignacio, E. H. , & Tumala, R. B. (2021). Professional quality of life and caring behaviours among clinical nurses during the COVID‐19 pandemic. Journal of Clinical Nursing, 1–13. 10.1111/jocn.15937 PMC8446991 34231269

[nop21676-bib-0037] Kaddourah, B. , Abu‐Shaheen, A. K. , & Al‐Tannir, M. (2018). Quality of nursing work life and turnover intention among nurses of tertiary care hospitals in Riyadh: A cross‐sectional survey. BMC Nursing, 17(1), 1–7. 10.1186/s12912-018-0312-0 30302056 PMC6167796

[nop21676-bib-0038] Kamal, A. H. , Bull, J. H. , Wolf, S. P. , Swetz, K. M. , Shanafelt, T. D. , Ast, K. , Kavalieratos, D. , & Sinclair, C. T. (2020). Prevalence and predictors of burnout among hospice and palliative care clinicians in the US. Journal of Pain and Symptom Management, 59(5), e6–e13. 10.1016/j.jpainsymman.2019.11.017 31778784

[nop21676-bib-0039] Keener, T. A. , Hall, K. , Wang, K. , Hulsey, T. , & Piamjariyakul, U. (2021). Relationship of quality of life, resilience, and associated factors among nursing faculty during COVID‐19. Nurse Educator, 46(1), 17–22. 10.1097/NNE.0000000000000926 32941307

[nop21676-bib-0040] Kelbiso, L. , Belay, A. , & Woldie, M. (2017). Determinants of quality of work life among nurses working in Hawassa town public health facilities, South Ethiopia: A cross‐sectional study. Nursing Research and Practice, 2017, 1–11. 10.1155/2017/5181676 PMC5742902 29379654

[nop21676-bib-0041] Koinis, A. , Giannou, V. , Drantaki, V. , Angelaina, S. , Stratou, E. , & Saridi, M. (2015). The impact of healthcare workers job environment on their mental‐emotional health. Coping strategies: The case of a local general hospital. Health Psychology Research, 3(1), 1984. 26973958 10.4081/hpr.2015.1984 PMC4768542

[nop21676-bib-0042] Labrague, L. J. , & de Los Santos, J. A. A. (2021). Fear of Covid‐19, psychological distress, work satisfaction and turnover intention among frontline nurses. Journal of Nursing Management, 29(3), 395–403. 10.1111/jonm.13121 32985046 PMC7537256

[nop21676-bib-0043] Lee, H.‐F. , Yen, M. , Fetzer, S. , & Chien, T. W. (2015). Predictors of burnout among nurses in Taiwan. Community Mental Health Journal, 51(6), 733–737. 10.1007/s10597-014-9818-4 25536942

[nop21676-bib-0044] Lorente, L. , Vera, M. , & Peiró, T. (2021). Nurses stressors and psychological distress during the COVID‐19 pandemic: The mediating role of coping and resilience. Journal of Advanced Nursing, 77(3), 1335–1344. 10.1111/jan.14695 33210768 PMC7753515

[nop21676-bib-0045] Mason, D. J. , Leavitt, J. K. , & Chaffee, M. W. (2013). Policy and Politics in Nursing and Healthcare‐Revised Reprint. Elsevier Health Sciences.

[nop21676-bib-0046] McFadden, P. , Ross, J. , Moriarty, J. , Mallett, J. , Schroder, H. , Ravalier, J. , Manthorpe, J. , Currie, D. , Harron, J. , & Gillen, P. (2021). The role of coping in the wellbeing and work‐related quality of life of UK health and social care workers during COVID‐19. International Journal of Environmental Research and Public Health, 18(2), 815. 10.3390/ijerph18020815 33477880 PMC7832874

[nop21676-bib-0047] McMurray, A. (2020). Healthy communities: The evolving roles of nursing. In Contexts of nursing 6 e (pp. 263–284). University of the Sunshine Coast.

[nop21676-bib-0048] Miles, J. M. , & Scott, E. S. (2019). A new leadership development model for nursing education. Journal of Professional Nursing, 35(1), 5–11. 10.1016/j.profnurs.2018.09.009 30709465

[nop21676-bib-0049] Mo, Y. , Deng, L. , Zhang, L. , Lang, Q. , Liao, C. , Wang, N. , Qin, M. , & Huang, H. (2020). Work stress among Chinese nurses to support Wuhan in fighting against COVID‐19 epidemic. Journal of Nursing Management, 28(5), 1002–1009. 10.1111/jonm.13014 32255222 PMC7262235

[nop21676-bib-0050] Murat, M. , Köse, S. , & Savaşer, S. (2021). Determination of stress, depression and burnout levels of front‐line nurses during the COVID‐19 pandemic. International Journal of Mental Health Nursing, 30(2), 533–543. 10.1111/inm.12818 33222350 PMC7753629

[nop21676-bib-0051] Nayak, T. , & Sahoo, C. K. (2015). Quality of work life and organizational performance: The mediating role of employee commitment. Journal of Health Management, 17(3), 263–273. 10.1177/0972063415589236

[nop21676-bib-0052] Ofei, A. M. , Paarima, Y. , Barnes, T. , & Kwashie, A. A. (2020). Stress and coping strategies among nurse managers. Journal of Nursing Education and Practice, 10(2), 39–48. 10.5430/jnep.v10n2p39

[nop21676-bib-0053] Organization, W. H . (2020). Mental health and psychosocial considerations during the COVID‐19 outbreak . World Health Organization. https://apps.who.int/iris/handle/10665/331490

[nop21676-bib-0054] Pereira, H. , Feher, G. , Tibold, A. , Costa, V. , Monteiro, S. , & Esgalhado, G. (2021). Mediating effect of burnout on the association between work‐related quality of life and mental health symptoms. Brain Sciences, 11(6), 813. 10.3390/brainsci11060813 34205291 PMC8235172

[nop21676-bib-0055] Poku, C. A. , Donkor, E. , & Naab, F. (2020). Determinants of emotional exhaustion among nursing workforce in urban Ghana: A cross‐sectional study. BMC Nursing, 19(1), 1–10. 10.1186/s12912-020-00512-z 33372600 PMC7722335

[nop21676-bib-0056] Poku, C. A. , Donkor, E. , & Naab, F. (2022). Impacts of nursing work environment on turnover intentions: The mediating role of burnout in Ghana. Nursing Research and Practice, 2022, 1–9. 10.1155/2022/1310508 PMC8898860 35265373

[nop21676-bib-0057] Pourteimour, S. , Yaghmaei, S. , & Babamohamadi, H. (2021). The relationship between mental workload and job performance among Iranian nurses providing care to COVID‐19 patients: A cross‐sectional study. Journal of Nursing Management, 29(6), 1723–1732. 10.1111/jonm.13305 33690932 PMC8236996

[nop21676-bib-0058] Rabacal, J. , Oducado, R. M. , & Tamdang, K. (2020). COVID‐19 impact on the quality of life of teachers: A cross‐sectional study. Asian Journal for Public Opinion Research, 8(4), 478–492. 10.15206/ajpor.2020.8.4.478

[nop21676-bib-0059] Roth, C. , Berger, S. , Krug, K. , Mahler, C. , & Wensing, M. (2021). Internationally trained nurses and host nurses' perceptions of safety culture, work‐life‐balance, burnout, and job demand during workplace integration: A cross‐sectional study. BMC Nursing, 20(1), 1–15. 10.1186/s12912-021-00581-8 33993868 PMC8127287

[nop21676-bib-0060] Saygili, M. , Avci, K. , & Sönmez, S. (2020). Quality of work life and burnout in healthcare Workers in Turkey. Journal of Health Management, 22(3), 317–329. 10.1177/0972063420938562

[nop21676-bib-0061] Schaufeli, W. B. , & Taris, T. W. (2014). A critical review of the job demands‐resources model: Implications for improving work and health. Bridging occupational, organizational and public health (pp. 43–68). Springer. 10.1007/978-94-007-5640-3_4

[nop21676-bib-0062] Scruth, E. A. , Garcia, S. , & Buchner, L. (2018). Work life quality, healthy work environments, and nurse retention. Clinical Nurse Specialist, 32(3), 111–113. 10.1097/NUR.0000000000000376 29621103

[nop21676-bib-0063] Shahrour, G. , & Dardas, L. A. (2020). Acute stress disorder, coping self‐efficacy and subsequent psychological distress among nurses amid COVID‐19. Journal of Nursing Management, 28(7), 1686–1695. 10.1111/jonm.13124 32767827 PMC7436502

[nop21676-bib-0064] Shakeri, F. , Atashzadeh‐Shoorideh, F. , Varzeshnejad, M. , Svetic Cisic, R. , & Oomen, B. (2021). Correlation between ethical intelligence, quality of work life and caring behaviour of Paediatric nurses. Nursing Open, 8(3), 1168–1174. 10.1002/nop2.729 34482658 PMC8046053

[nop21676-bib-0065] Sheroun, D. , Wankhar, D. D. , Devrani, A. , Lissamma, P. V. , & Chatterjee, K. (2020). A study to assess the perceived stress and coping strategies among B. Sc. Nursing students of selected colleges in Pune during COVID‐19 pandemic lockdown. International Journal of Science and Healthcare Research, 5(2), 280–288.

[nop21676-bib-0066] Shoja, E. , Aghamohammadi, V. , Bazyar, H. , Moghaddam, H. R. , Nasiri, K. , Dashti, M. , Choupani, A. , Garaee, M. , Aliasgharzadeh, S. , & Asgari, A. (2020). Covid‐19 effects on the workload of Iranian healthcare workers. BMC Public Health, 20(1), 1–7. 10.1186/s12889-020-09743-w 33138798 PMC7605333

[nop21676-bib-0067] Suleiman, K. , Hijazi, Z. , Al Kalaldeh, M. , & Sharour, L. A. (2019). Quality of nursing work life and related factors among emergency nurses in Jordan. Journal of Occupational Health, 61(5), 398–406. 10.1002/1348-9585.12068 31215754 PMC6718837

[nop21676-bib-0068] Warshawsky, N. , & Cramer, E. (2019). Describing nurse manager role preparation and competency: Findings from a national study. The Journal of Nursing Administration, 49(5), 249–255. 10.1097/NNA.0000000000000746 30973429

[nop21676-bib-0069] Wei, H. , Sewell, K. A. , Woody, G. , & Rose, M. A. (2018). The state of the science of nurse work environments in the United States: A systematic review. International Journal of Nursing Sciences, 5(3), 287–300. 10.1016/j.ijnss.2018.04.010 31406839 PMC6626229

[nop21676-bib-0070] Xu, H. G. , Kynoch, K. , Tuckett, A. , Eley, R. , & Newcombe, P. (2019). Effectiveness of interventions to reduce occupational stress among emergency department staff: A systematic review protocol. JBI Database of Systematic Reviews and Implementation Reports, 17(4), 513–519. 10.11124/JBISRIR-2017-003955 30973525

